# Durvalumab and tremelimumab before surgery in patients with hormone receptor positive, HER2-negative stage II–III breast cancer

**DOI:** 10.18632/oncotarget.28567

**Published:** 2024-03-19

**Authors:** Haven R. Garber, Sreyashi Basu, Sonali Jindal, Zhong He, Khoi Chu, Akshara Singareeka Raghavendra, Clinton Yam, Lumarie Santiago, Beatriz E. Adrada, Padmanee Sharma, Elizabeth A. Mittendorf, Jennifer K. Litton

**Affiliations:** ^1^Department of Breast Medical Oncology, University of Texas MD Anderson Cancer Center, Houston, TX 77030, USA; ^2^Department of Immunology, University of Texas MD Anderson Cancer Center, Houston, TX 77030, USA; ^3^Department of Breast Imaging, University of Texas MD Anderson Cancer Center, Houston, TX 77030, USA; ^4^Department of Genitourinary Medical Oncology, University of Texas MD Anderson Cancer Center, Houston, TX 77030, USA; ^5^Department of Surgery, Division of Breast Surgery, Brigham and Women’s Hospital, Boston, MA 02115, USA; ^6^Breast Oncology Program, Dana-Farber Brigham Cancer Center, Boston MA 02115, USA; ^7^Harvard Medical School, Boston, MA 02115, USA

**Keywords:** breast cancer, ER positive, immunotherapy, neoadjuvant chemotherapy, tumor microenvironment

## Abstract

A clinical trial was conducted to assess the feasibility of enrolling patients with Stage II or III hormone receptor positive (HR+)/HER2-negative breast cancer to pre-operative dual PD-L1/CTLA-4 checkpoint inhibition administered prior to neoadjuvant chemotherapy (NACT). Eight eligible patients were treated with upfront durvalumab and tremelimumab for two cycles. Patients then received NACT prior to breast surgery. Seven patients had baseline and interval breast ultrasounds after combination immunotherapy and the responses were mixed: 3/7 patients experienced a ≥30% decrease in tumor volume, 3/7 a ≥30% increase, and 1 patient had stable disease. At the time of breast surgery, 1/8 patients had a pathologic complete response (pCR). The trial was stopped early after 3 of 8 patients experienced immunotherapy-related toxicity or suspected disease progression that prompted discontinuation or a delay in the administration of NACT. Two patients experienced grade 3 immune-related adverse events (1 with colitis, 1 with endocrinopathy). Analysis of the tumor microenvironment after combination immunotherapy did not show a significant change in immune cell subsets from baseline. There was limited benefit for dual checkpoint blockade administered prior to NACT in our study of 8 patients with HR+/HER2-negative breast cancer.

## INTRODUCTION

The clinical efficacy of immune checkpoint inhibitors has been disappointing for metastatic hormone receptor positive (HR+)/HER2/Neu (HER2)-negative breast cancer, the most common subtype of the disease. While there is clinical benefit for combined anti-PD-1 blockade and chemotherapy for a subset of patients with early stage [1, 2] and metastatic triple negative breast cancer [3, 4], no such gains have been observed for most patients with HR+/HER2-negative breast cancer, particularly in the metastatic setting. For example, in KEYNOTE-028, 25 patients with PD-L1+ (combined positive score, (CPS) ≥1), HR+/HER2-negative metastatic breast cancer were treated with single-agent pembrolizumab, resulting in an overall response rate (ORR) of 12% [5]. In the phase Ib JAVELIN trial, 72 patients with HR+/HER2-negative metastatic breast cancer were treated with single-agent avelumab (anti-PD-L1) and the ORR was 2.8% [6]. Tolaney et al. evaluated the combination of eribulin plus pembrolizumab versus eribulin alone in a randomized trial that included 88 patients with HR+/HER2-negative metastatic breast cancer. No difference was observed in ORR, progression-free survival (PFS), or overall survival (OS) between the two groups [7]. Notably, the patients in these 3 trials had received multiple lines of prior therapy. In contrast, the phase III KEYNOTE-756 trial randomized 1278 untreated patients with high-risk, early-stage HR+/HER2-negative breast cancer to receive either pre-operative pembrolizumab and chemotherapy or pre-operative placebo and chemotherapy prior to breast surgery. An improved rate of pCR was seen in the pembrolizumab group compared to placebo (24.3% vs. 15.6%), though data regarding event-free survival remain immature [8]. A second neoadjuvant trial, CheckMate 7FL, also reported improved pCR with the addition of nivolumab to chemotherapy for patients with high-risk HR+/HER2-negative early breast cancer [9]. These results suggest that early-stage patients with HR+/HER2-negative breast cancer may have tumors that are more susceptible to immunotherapy.

In addition to treating patients with earlier stage disease, another strategy that can increase the response rate to immunotherapy is dual PD-(L)1/CTLA-4 checkpoint inhibition. Combined ipilimumab/nivolumab immune therapy has gained FDA approval across multiple tumor types in the advanced setting, including melanoma, renal cell carcinoma, microsatellite instability-high or mismatch repair deficient colorectal cancer, hepatocellular carcinoma, non-small cell lung cancer, and mesothelioma. Combined PD-1/CTLA-4 blockade promotes T cell infiltration into tumors [10, 11]. Increased tumor-infiltrating lymphocytes (TILs) are associated with improved responses to neoadjuvant chemotherapy (NACT) across breast cancer subtypes [12]. Therefore, we hypothesized that amplifying TIL via dual checkpoint blockade would enhance the response to subsequent NACT in breast tumors [10–12]. In the present study, we assessed the feasibility of enrolling untreated patients with stage II or III HR+/HER2-negative breast cancer to upfront experimental treatment with combined PD-L1/CTLA-4 checkpoint inhibition prior to standard NACT and surgery. Patient tumor samples were collected to assess immunologic and molecular responses to combination checkpoint blockade.

## RESULTS

Patients participated in the study beginning May of 2018 with the last patient starting treatment in January of 2019. Eight patients enrolled and received at least 1 cycle of investigative combination immunotherapy with preoperative durvalumab and tremelimumab. Patient characteristics are shown in Table 1.

**Table 1:** Clinical characteristics of patients treated with pre-operative combination immunotherapy

Age at diagnosis		Clinical T stage	
Median 55	(range 47–66)	T2	7 (87.5%)
**Gender**		T3	1 (12.5%)
Female	8 (100.0%)	**Clinical *N* stage**	
**Race**		N0	2 (25.0%)
Asian	1 (12.5%)	N1	6 (75.0%)
White	7 (87.5%)	**Clinical prognostic stage**	
**Ethnic group**		Stage IIA	6 (75.0%)
Non-Hispanic or non-Latino	8 (100.0%)	Stage IIB	2 (25.0%)
**Menopausal status**		**# of IO cycles delivered**	
Pre-menopausal	3 (37.5%)	1	3 (37.5%)
Post-menopausal	5 (62.5%)	2	5 (62.5%)
**Histology**		**Timing of post-IO ddAC/paclitaxel**	
IDC	5 (62.5%)	Neoadjuvant	6 (75%)
IDC w/lobular features	2 (25.0%)	Adjuvant	1 (12.5%)
IDC w/mucinous features	1 (12.5%)	None	1 (12.5%)
**Nottingham grade**		**Breast surgery**	
1	1 (12.5%)	Segmental mastectomy	5 (62.5%)
2	4 (50.0%)	Mastectomy	1 (12.5%)
3	3 (37.5%)	Bilateral mastectomy	1 (12.5%)
**Ki-67**		**Adjuvant radiation**	
Low (<17%)	2 (25.0%)	Yes	7 (87.5%)
Moderate (17–35%)	3 (37.5%)	No	1 (12.5%)
High (> 35%)	1 (12.5%)	**Residual disease at surgery**	
Not done	2 (25.0%)	pCR	1 (12.5%)
**Receptor subtype at diagnosis**		ypT1c(m), ypN1mi	1 (12.5%)
ER pos/PR pos/HER2 neg	7 (87.5%)	ypT2, ypN1a	3 (37.5%)
ER pos/PR low pos/HER2 neg	1 (12.5%)	ypT2, ypN2a	2 (25%)
		ypT3, ypN2a	1 (12.5%)

**Figure 1:**
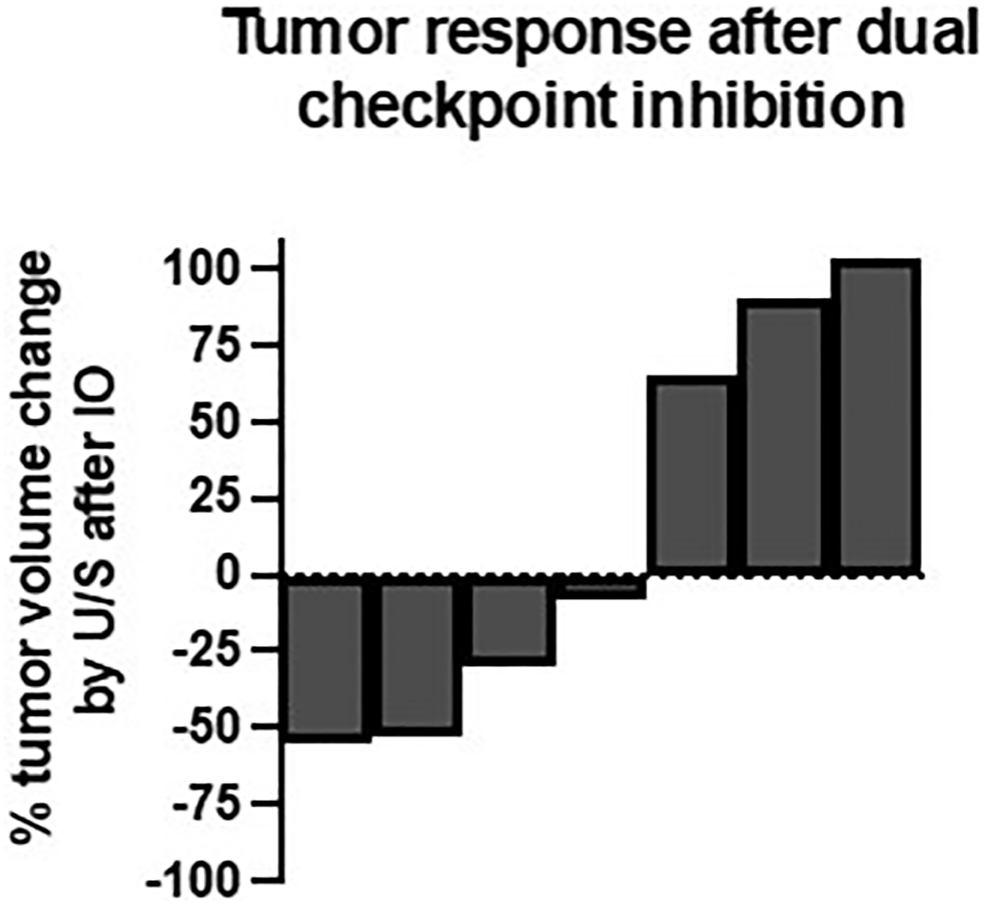
Tumor response after dual checkpoint inhibition. Change in breast tumor volume by ultrasound measurement from baseline to 1–2 cycles after immunotherapy (*n* = 7).

Seven patients had a baseline and interval breast ultrasound after immunotherapy. The percent change in tumor volume is shown in Figure 1. Three patients had a 30% or more reduction in primary tumor volume after 1 or 2 cycles of immunotherapy. Three patients had a more than 30% increase in tumor volume after 1 or 2 cycles of combination immunotherapy and one patient’s tumor changed by 8%, essentially stable disease. Patient 2, whose tumor was grade 3 with a high Ki67 and high Oncotype DX score, received only 1 cycle of durvalumab and tremelimumab and then reported an increase in the size of her primary tumor. This was corroborated by ultrasound (+104%), which showed increased internal vascularity within the mass, a possible satellite tumor, and increased size of a previously biopsied benign level I axillary lymph node. Of note, repeat biopsy of the axillary lymph node and biopsy of the possible satellite mass after the 1 cycle of combination immunotherapy were benign. It is difficult to differentiate tumor progression from pseudoprogression in this patient’s case since a repeat biopsy of the index breast tumor was not performed prior to NACT. This patient was the sole participant to experience a pCR at the time of breast surgery after NACT. The other participants all had residual disease in the breast and lymph nodes on final surgical pathology.

Two of 8 patients experienced grade 3 immunotherapy-related toxicity. One patient developed symptoms of colitis a week after the first cycle of durvalumab and tremelimumab. Her symptoms flared when a steroid dose taper was attempted, and she was ultimately hospitalized and treated with vedolizumab with improvement. The patient was unable to receive the planned NACT due to colitis and was instead treated with pre-operative endocrine therapy followed by breast surgery four months after the cycle of investigative immunotherapy. She received standard chemotherapy in the adjuvant setting. A second patient developed neck pain and swelling and body aches 2 weeks after the first cycle of durvalumab and tremelimumab. She was diagnosed with acute thyroiditis and adrenal insufficiency during a subsequent hospitalization and improved with thyroid hormone replacement and hydrocortisone, which she has required long-term. She subsequently declined systemic chemotherapy and underwent breast surgery two months after the cycle of investigative immunotherapy. Overall, rash and mucositis were the most common toxicities. The full list of recorded toxicities is listed in Table 2. All other reported toxicities were grade 1 or 2.

**Table 2:** Adverse events during neoadjuvant IO (*n* = 8 patients)

Treatment-related adverse event	Any grade	Grade 3/4
Adrenal insufficiency	1 (12.5%)	1 (12.5%)
Colitis	1 (12.5%)	1 (12.5%)
Conjunctivitis	1 (12.5%)	
Constipation	1 (12.5%)	
Diarrhea	1 (12.5%)	
Dysgeusia	1 (12.5%)	
Hot flashes	1 (12.5%)	
Hyperthyroidism	1 (12.5%)	1 (12.5%)
Mucositis	2 (25%)	
Pruritis	1 (12.5%)	
Rash	2 (25%)	

**Figure 2:**
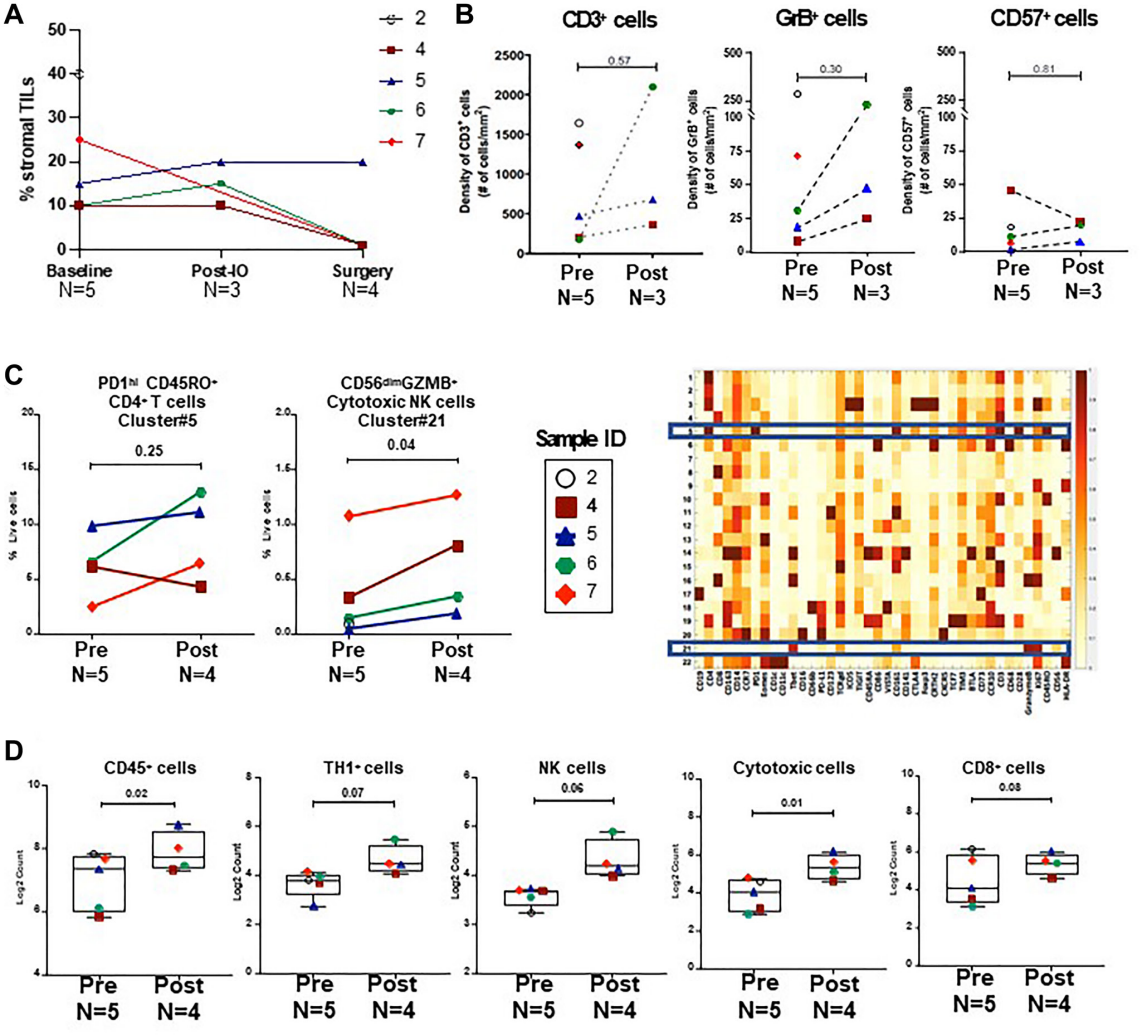
Analysis of immune cell subsets during neoadjuvant therapy. (**A**) Stromal TIL at baseline, post-immunotherapy, and post-NACT (surgical pathology) timepoints. (**B**) Quantitative IHC analysis for CD3^+^, GrB^+^ and CD57^+^ cells pre (*n* = 5) and post (*n* = 3) durvalumab and tremelimumab. (**C**) CyTOF analysis of CD45^+^ tumor infiltrating immune cells in pre (*n* = 5) and post-durvalumab/tremelimumab treated (*n* = 4) tumor samples. Unsupervised clustering was performed on CyTOF data of CD45^+^ cells, using the PhenoGraph algorithm and cluster frequencies were plotted for PD1^hi^ ICOS^+^ CD4^+^ effector memory T cells (cluster#5) and CD56^dim^ cytotoxic NK cells (cluster#21) in the corresponding heatmap of the relative expression levels of all 37 proteins. (**D**) NanoString gene expression analysis was performed on pre (baseline) (*n* = 5) and post-NACT (*n* = 4) tumor tissue samples. Box plots show the distribution of immune scores (log2 normalized counts) of CD45^+^ cells, TH1 cells, NK cells, cytotoxic cells, and CD8^+^ T cells. *P*-values were calculated using two-tailed paired Student’s *t* test for all analysis.

The tumor microenvironment (TME) was examined using H&E, IHC, mass cytometry by time-of-flight (CyTOF), and NanoString nCounter gene expression analyses to evaluate immunologic responses to combination therapy with durvalumab and tremelimumab and to NACT. Stromal TIL were evaluated at baseline, post-immunotherapy, and post-NACT (surgical pathology) timepoints and remained stable after 2 cycles of dual checkpoint blockade in the three patients with serial biopsies available (Figure 2A). IHC characterization of immune cell subsets showed no difference in CD3^+^ T cells (the majority of which were CD4^+^ T cells), Granzyme B^+^ activated lymphocytes, or CD57^+^ natural killer cells after combination immunotherapy when compared to pretreatment tissue samples (*n* = 5, Figure 2B, Supplementary Figure 1A).

To phenotype tumor infiltrating leukocytes in further detail, we performed CyTOF analysis on fresh tumor tissue samples collected after 2 cycles of durvalumab and tremelimumab therapy and compared to pre-treatment tissue samples. We did not observe a significant difference in the immune infiltrate after combination immunotherapy, including in ICOS^+^ CD4^+^ T cells (Cluster #5), which represent a potential biomarker for biologic activity of anti-CTLA-4 therapy (Figure 2C). There was a trend towards an increase in activated natural killer cells (Cluster #21) after dual checkpoint inhibition, however these differences were not significant when accounting for testing multiple hypotheses.

Further study of the TME from post-NACT samples by gene expression analysis (NanoString) indicated significant increases in cell type scores for immune cells (CD45^+^) and cytotoxic cells by paired *t*-test in tumors when compared to pre-treatment samples (Figure 2D). However, in IHC analyses, no increase in CD57^+^ or GrB+ cells was observed in post-NACT samples when compared to baseline (Supplementary Figure 1B).

## DISCUSSION

This feasibility study was conducted to begin testing the hypothesis that dual checkpoint blockade would increase TIL and enhance the response to subsequent NACT in patients with stage II or III HR+/HER2-negative breast cancer. The trial’s target accrual was 16 patients; however, it was stopped early after 3 of the first 8 enrolled patients experienced immunotherapy-related toxicity or suspected disease progression indicating that this strategy is not clinically feasible. Among the 8 patients that did receive the study-specified combination immunotherapy, seven had pre- and post-immunotherapy ultrasounds performed showing the responses were mixed with 3 experiencing an increase in tumor volume, 3 experiencing a decrease in tumor volume, and one showing stable disease. The impact of combination immunotherapy on TIL was also mixed. Though limited by the number of patients with available serial biopsies, there did not appear to be a significant increase in the immune response within the TME.

The Phase II NIMBUS trial also assessed dual checkpoint blockade in breast cancer, though in a population of metastatic breast cancer patients with tumors harboring a high tumor mutation burden (TMB 9 mutations per megabase (mut/Mb)) [13]. Of the 30 patients enrolled, 20 had ER+/HER2-negative breast cancer. The ORR was 5/30 (16.7%) with 4 durable responses lasting at least 15 months. Three of the five responders had a tumor mutation burden TMB ≥14 Mut/Mb. The ORR among patients with TMB <14 Mut/Mb was 2/30 (6.7%). Three patients (10%) experienced grade 3 immune toxicity. Though we did not measure TMB in our patients, probabilistically the majority were TMB-low [14]. The TAPUR basket trial similarly included patients with TMB-high metastatic breast cancer but utilized single agent anti-PD-1 checkpoint blockade (pembrolizumab) rather than combination immunotherapy [15]. Half of the 28 enrolled patients had ER+ breast cancer and the majority had received multiple prior lines of systemic therapy. The ORR was 21% with a median PFS of 10.6 weeks. Five patients (17.9%) experienced one or more grade 3 adverse events that were possibly attributed to pembrolizumab and 6 patients discontinued treatment due to side effects. In summary, a minority of patients with ER+ metastatic breast cancer may benefit from anti-PD-(L)1 anti-CTLA-4 checkpoint blockade, however, the majority risk exposure to immune related adverse events without additional benefit.

Patients in the combination anti-PD1 and chemotherapy arm of KEYNOTE-756 showed a pCR rate of 24.3% (versus 1/8 (12.5%) in our small cohort) [8]. The KEYNOTE-756 protocol differed from ours in several ways including the concurrent delivery of immunotherapy with chemotherapy versus sequentially in our trial, pembrolizumab vs. durvalumab/tremelimumab as immune therapy, and the inclusion of only patients with high grade tumors in KEYNOTE-756 compared with our study that included patients regardless of tumor grade. The KEYNOTE 756 and Checkmate 7FL trials will help clarify the potential benefit of adding single agent anti-PD-1 checkpoint blockade to NACT for patients with high-risk HR+/HER2-negative, Stage II/III breast cancer [8, 9]. The trials will also be carefully analyzed for the risk of severe and/or long-term toxicity because most patients with HR+/HER2-negative early-stage breast cancer are cured with standard therapy. For this reason, the risk/benefit calculus of adding immunotherapy for HR+/HER2-negative early breast cancer is different from metastatic triple negative breast cancer (TNBC) or even Stage II/III TNBC where the risks of morbidity and mortality from disease are higher. Hopefully, biomarkers such as PD-1 and TMB will guide the use of single or dual agent immunotherapy towards those patients most likely to benefit, sparing others from significant toxicity. Notably, immune-mediated adverse events of grade 3 or higher were reported in 12.9% of breast cancer patients receiving pembrolizumab in the KEYNOTE-522 trial and in 38% of patients receiving dual ipilimumab/nivolumab in a trial of patients with metastatic melanoma [1, 16].

Analysis of the TME in a limited number of serial breast cancer biopsies from patients treated on our protocol did not show a significant change in the immune TME after 2 cycles of durvalumab and tremelimumab. There are likely several explanations for this finding including the small sample size, the timing of the post-immunotherapy biopsy (very shortly after cycle 2), the difficulty in preparing single cell suspensions from breast tumors (CyTOF), and the inclusion of several patients with low or moderate grade HR+ breast cancer, a tumor biology that can be less responsive to immunotherapy.

A less favorable immune TME is believed to account for the lower clinical activity of immune checkpoint blockade in HR+ breast cancer compared to TNBC. Expression of PD-L1 is generally lower in HR+ tumors than in TNBC [17, 18]. The number of TIL, an important prognostic biomarker in early stage TNBC, is also lower in HR+ breast cancer [12, 19]. Lastly, TMB is lower in HR+/HER2-negative breast cancer than in TNBC [14]. Despite this overarching theme, HR+/HER2-negative breast is a heterogeneous disease and the immune infiltrate is similarly variable [20, 21].

The main limitation of our study was its small size with only 8 patients evaluated. The trial was stopped prior to enrollment of the planned 16 patients due to 3 of 8 (37.5%) patients experiencing potential harm in the form of immunotherapy-related toxicity and/or a treatment delay. The toxicity of dual checkpoint blockade is well-described, and it is unlikely that patients with early stage breast cancer are more susceptible to immunotherapy-related toxicity than patients with other tumor types, though there is evidence that sex plays a role [16, 22]. Another limitation to our study was the lack of uniform disease biology. Though all 8 patients had HR+/HER2-negative disease, the breast tumors exhibited disparate disease characteristics, including in tumor grade, Ki67, and histology. Certain biologic subsets of HR+/HER2-negative breast cancer (e.g., grade 3 tumors or high genomic assay scores) may be more susceptible to checkpoint blockade. Nevertheless, our trial was novel in that few studies have assessed dual checkpoint inhibition in the most common breast cancer subtype, HR+/HER2-negative, early-stage breast cancer. Our study also included a careful investigation of the TME.

In conclusion, this trial was stopped early after 3 of the first 8 enrolled patients experienced immunotherapy-related toxicity or suspected disease progression indicating that this strategy is not clinically feasible in this patient population. In the small cohort of 8 patients with HR+/HER2-negative breast cancer, there was limited benefit for dual checkpoint blockade administered prior to NACT. Only one patient (12.5%) experienced a pCR after immune therapy and NACT. Two patients experienced grade 3 immunotherapy-related toxicity. For immunotherapy to play a meaningful role in HR+/HER2 negative early breast cancer, a breast cancer subtype where most patients are cured with standard therapy, it will need to significantly increase the fraction of cured patients without disproportionately causing serious and/or long-term immune toxicity.

## METHODS

### Clinical trial

This was a pilot study that aimed to accrue 16 patients to evaluate the feasibility of enrolling patients with clinical stage II or III HR+/HER2-negative breast cancer onto a trial evaluating investigational immunotherapy agents prior to standard NACT. Durvalumab was administered at a dose of 1500 mg IV and tremelimumab at a dose of 75 mg IV for 2 cycles on days 1 and 28. Patients then proceeded to standard NACT followed by breast surgery. In addition to feasibility of enrollment, another primary objective was to evaluate the safety and tolerability of durvalumab and tremelimumab. The secondary objective was to assess the immunologic and molecular responses to durvalumab and tremelimumab. To be eligible, patients had to have HR+/HER2-negative breast cancer. HR+ was defined as estrogen receptor (ER) and/or progesterone receptor (PR) expression >10% by immunohistochemistry (IHC) and HER2-negative was defined as 0/1+ by IHC or if 2+, negative by fluorescence *in situ* hybridization according to ASCO-CAP criteria. Other inclusion criteria included ECOG performance status of 0 or 1, planned NACT, and adequate blood counts and organ function. Patients were excluded if they had received prior PD-1, PD-L1, or CTLA-4 inhibitors or any prior treatment for the primary breast cancer. Other exclusion criteria included: current or prior use of immunosuppressive medications within 28 days (not to exceed 10 mg/day of prednisone or equivalent steroid), active or previous autoimmune disease within 2 years, inflammatory bowel disease, or receipt of a live attenuated vaccination within 30 days prior to study entry or prior to study treatment. All patients who received at least 1 cycle of investigative immunotherapy were considered evaluable. Toxicities were monitored and recorded per the Common Terminology Criteria for Adverse Events version 4.03. Toxicities were reported with the highest grade observed per individual. This trial was conducted under an institutional review board-approved protocol 2016-0902 and in accordance with relevant guidelines at The University of Texas MD Anderson Cancer Center. Informed consent was obtained from each participant.

### Correlative studies

#### Imaging and biospecimen collection

Baseline breast ultrasounds were performed within 21 days before the first cycle of investigative immunotherapy and again between 1 and 7 days after the second cycle. Research biopsies were collected at baseline and after 2 cycles of investigative immunotherapy.

#### TIL assessment/immunohistochemistry

Hematoxylin and eosin (H&E) staining, and IHC were performed on 4 μm formalin-fixed paraffin-embedded (FFPE) pre- and post-treatment tissue samples. Stromal TILs were quantified manually by H&E-stained FFPE sections and scored as a percentage of stromal area according to the International Immuno-Oncology Working Group method for assessing TILs [23]. IHC staining on subsequent sections was performed using anti-CD4 (Abcam, cat#ab133616, 1:250), anti-Gr-B (11F1) (Leica Microsystems, cat#PA0291, ready-to-use), and anti-CD57 (BD Biosciences, cat# 347390, 1:40) antibodies. Sections were stained using the BondRX instrument and the Bond Polymer Refine Detection kit (Cat. #DS9800), and the IHC slides were scanned and digitized using the ScanscopeXT system (Aperio/Leica Technologies). Single stain IHC quantification analysis was performed by the pathologist using the HALO 2.3.2089.70 software (Indica Labs). The number of marker positive cells for each analysis area were calculated and expressed as density (number of positive cells/mm2) and densities were plotted using Prism V8.4.3 (GraphPad). Statistical analysis was done using a two-tailed, paired *t*-test, and *P* < 0.05 was considered statistically significant.

#### Mass cytometry

Fresh tumor tissue was dissociated with GentleMACS system (Miltenyi Biotec; Bergisch Gladbach, Germany) as per the manufacturer’s instructions. Single cell suspensions were stained with 37 antibodies (CD45, CD8a, CD57, PD1 (CD279), CD38, CD4, CD11c, Tbet, CD16, CD66b, PD-L1 (CD274), CD123, ICOS (CD278), CD326, CD163, CD45RA, CD86, CD33, VISTA, CD14, EOMES, FOXP3, CD119, CD15, CD40, TIM3 (CD366), CD11b, CD206, CD19, CD3, CD68, PD-L2 (CD273), CD115 (CSF1R), Ki67, CD45RO, CD56, HLA-DR), cisplatin, and Ir DNA-Intercalator. Antibodies were either purchased pre-conjugated from Fluidigm or purchased purified and conjugated using MaxPar X8 Polymer kits (Fluidigm) according to the manufacturer’s instructions. Briefly, samples were stained with cell-surface antibodies in phosphate-buffered saline (PBS) containing 5% goat serum and 1% bovine serum albumin (BSA) for 30 minutes at 4°C. Optimal antibody concentrations were determined by serial dilution staining of human peripheral blood mononuclear cells. After viability staining with 25 μM cisplatin (Fluidigm) in PBS containing 1% BSA, samples were washed in PBS containing 1% BSA, fixed and permeabilized according to manufacturers’ instructions using the FoxP3 staining buffer set (eBioscience) before being incubated with intracellular antibodies in permeabilization buffer for 30 min at 4°C. Samples were washed and incubated in Ir intercalator (Fluidigm) and stored at 4°C until acquisition, generally within 12 hours. Right before acquisition samples were washed and re-suspended in water containing EQ 4 element beads (Fluidigm). Samples were acquired on a Helios mass cytometer (Fluidigm).

#### Mass cytometry analysis

Files (fcs) were normalized using a bead-based normalization software for mass cytometry data (R package premessa, Parker Institute for Cancer Immunotherapy) [24]. Samples were then manually gated in FlowJo by event length, by live/dead discrimination, and for populations of interest using lineage markers (CD45) for separate analyses. Data were then exported into MATLAB as fcs files for downstream analysis and arcsinh transformed using a coefficient of 5 (x_transformed = arsinh(x/5)). To visualize the high-dimensional data in two dimensions, the t-Distributed Stochastic Neighbor Embedding (t-SNE) dimension reduction algorithm [25] was applied to the cells, using all channels besides those used to manually gate the population of interest (e.g., CD45).

#### Mass cytometry clustering

Clustering analysis was performed using the MATLAB implementation of the PhenoGraph clustering algorithm [26]. Clusters were identified using PhenoGraph on all the samples in the space formed by these principal components, with the parameter k for the number of nearest neighbors selected uniquely for each sample using the formula k = minimum (0.002^*^ number of cells, 10). PhenoGraph was run with parameter k = 100, resulting in 22 clusters in the CD45^+^ analysis.

#### NanoString

FFPE tumor tissue sections were evaluated for tumor content and tissue quality by pathology. Tissue blocks which were approved by pathology were processed for RNA isolation by de-waxing using deparaffinization solution (QIAGEN, Valencia, CA, USA). Total RNA was extracted using the RecoverALL Total Nucleic Acid Isolation kit (Ambion, Austin, TX) according to the manufacturer’s instructions. RNA purity was assessed on the ND-Nanodrop1000 spectrometer (Thermo Fisher Scientific, Wilmington, MA, USA). For NanoString assay, 100 ng of RNA was used to detect immune gene expression using nCounter PanCancer Immune Profiling panel along with custom CodeSet. Counts of the reporter probes were tabulated for each sample by the nCounter Digital Analyzer and raw data output was imported into nSolver (http://www.nanostring.com/products/nSolver, v.4.0). An nSolver data analysis package was used for normalization, cell type analysis and gene signature. The signature score was calculated as the median gene expression. Hierarchical clustering and heatmap analysis were performed with Qlucore Omics Explorer version 3.7 software (Qlucore, NY, USA). Data were plotted using GraphPad Prism 8 (GraphPad Software v-9) and two tailed Student’s *t* test was used to compare the means of 2 groups and *P*-values less than 0.05 were considered significant.

## SUPPLEMENTARY MATERIALS



## Abbreviations

CPS: Combined positive score
CyTOF: Mass cytometry by time-of-flight
FFPE: Formalin-fixed paraffin-embedded
HER2: HER2/Neu
HR+: Hormone receptor positive
IHC: Immunohistochemistry
NACT: Neoadjuvant chemotherapy
ORR: Overall response rate
OS: Overall survival
PBS: Phosphate buffered saline
pCR: Pathologic complete response
PFS: Progression free survival
TMB: Tumor mutation burden
TME: Tumor microenvironment
TNBC: Triple negative breast cancer
