# Supplementary material for: Durvalumab and tremelimumab before surgery in patients with hormone receptor positive, HER2-negative stage II–III breast cancer
Source: Oncotarget. 2024 Mar 19;15:238–47. doi: 10.18632/oncotarget.28567 (PMC10950364; doi:10.18632/oncotarget.28567)
Supplement: Supplementary file 1 [file oncotarget-15-28567-s001.pdf]

Durvalumab and tremelimumab before surgery in patients with hormone receptor positive, HER2-negative stage II–III breast cancer

SUPPLEMENTARY MATERIALS

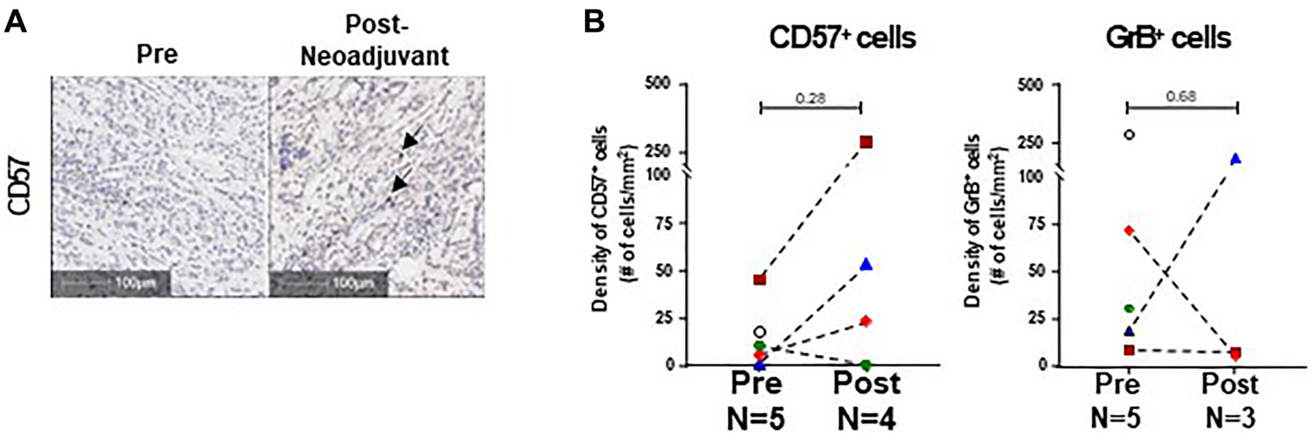

Supplemental Figure 1: Analysis of CD57+ and GrB+ immune cells during neoadjuvant therapy. (**A**) CD57 staining at pre and post-NACT timepoints (Patient 5). (**B**) Quantitative IHC analysis for CD57$^+$ and GrB$^+$ cells at baseline and post-NACT. *P*-values were calculated using two-tailed paired Student's *t* test for all analysis.
